# The Story of the Insane from Year to Year

**Published:** 1895-04-27

**Authors:** 


### THE STORY OF THE INSANE FROM YEAR TO YEAR.

(7th Series.)

### ROYAL ASYLUM, EDINBURGH.

The statistical tables show a decrease of eleven patients during the year, there being 843 on the books on January 31st. The admissions were 426, the recoveries 189, discharged relieved 106, and the deaths 110. Put into percentages, these numbers show that 44·4 per cent. of the admissions recovered, and 13 per cent. of the average number resident died. Both of these percentages exceed the average in asylums. The insanity in 22 of the year's admissions was caused by adverse circumstances and worry, in 81 by intemperance in drink, and in 129 by hereditary influence. Of the deaths, 64 were caused by brain diseases, and 12 by consumption. Dr. Clouston's report goes over a good deal of ground, and, as might be expected, all his remarks are interesting and pointed. When speaking of the popular notions of insanity he says most truly: "The feeling that it is in some way a disgrace to a family to have a member subject to this disease often makes the affliction ten times heavier to bear than it really need be. It is not faced up in the manful and candid way with which we meet our other misfortunes." No doubt Dr. Clouston is right as to the fact; but has not this very feeling of disgrace some little effect in preventing still more insanity in the future? Our efforts should lie far more in the direction of prevention than of cure. Indeed, he brings statistics in to show that half of our families are affected by idiotcy, epilepsy, or insanity. And further on he says: "Cure is the highest aim of the institution, and when that is attained we feel that the year's work has been to that extent at least well done." Yes, that is the physician's view of it; indeed, the only view the physician can take; but if Dr. Clouston were to investigate the results of the cures for the year 1863 at the Royal Asylum he would almost certainly find that these cures had tremendously increased the admissions for 1893, and the political economist would take a different view from the physician. The Commissioners in Lunacy say the asylum was everywhere found in excellent order, and the managers record their appreciation of the services of Dr. Clouston. The cost per head per annum of the paupers' maintenance was £30 7s. 3d., while that of the private patients ranges from £39 to £96.

### JOINT COUNTIES ASYLUM, CARMARTHEN.

On January 1st this asylum contained 556 patients, and by the end of the year the number had fallen to 546. There were 86 admissions, 26 recoveries, and 52 deaths. The percentage of recoveries stands at 32·5 on the admissions, and that of the deaths at 9·33, on the average number resident. Only five of the admissions are put down to drink, and only 16 to hereditary influence. Of the deaths 12 were due to cerebral diseases, and five to consumption. Owing to the illness of Dr. Hearder, the report is presented by Dr. Gibbon, the acting medical superintendent. The accommodation for patients is practically exhausted, there being only ten vacancies for men and nineteen for women, although the Hospital for Infectious Disease has been requisitioned, a course which could hardly be recommended. The committee say it "affords them pleasure to again speak in high terms of the efficient management of the institution, and of the valuable services which are rendered by their medical superintendent (Dr. Hearder) and his efficient staff." The weekly cost of maintenance is a fraction over 8s. 5d. per head.

